# Whale See, Whale Do: Drone Observations Reveal Social Learning Processes in Mom‐Calf Pairs

**DOI:** 10.1002/ece3.74517

**Published:** 2026-10-14

**Authors:** Clara N. Bird, Leigh G. Torres

**Affiliations:** ^1^ Geospatial Ecology of Marine Megafauna Lab, Department of Fisheries, Wildlife and Conservation Sciences, Marine Mammal Institute Oregon State University Newport Oregon USA; ^2^ Lab for Animal Behavioural Interaction Research in the Ocean, Department of Fisheries, Wildlife & Conservation Sciences, Marine Mammal Institute Oregon State University Newport Oregon USA

**Keywords:** baleen whales, behavior, drones, enhancement, imitation, mom‐calf, social learning

## Abstract

Social learning is a key indicator of culture and a valuable process to understand due to effects on behavioral diversity and specialization with subsequent impacts on population survival. While social learning has been identified and quantified across a diverse array of taxa, the specific underlying processes are rarely identified. However, identifying the mimetic process involved in learning is important as it informs our understanding of a species' cognitive abilities and contributes to the study of cultural and cognitive evolution. Social learning and culture are often studied in cetaceans, particularly amongst odontocetes, but processes have only been identified in human care. Here, we analyze opportunistic drone footage of Pacific Coast Feeding Group (PCFG) gray whale mom‐calf pairs foraging off the coast of Oregon, USA for evidence of foraging tactic mimicry and evaluate alignment with different mimetic processes. We filmed 15 pairs across 9 years and observed a calf performing a foraging tactic while its mom was foraging in 6 of the pairs. The calf always initiated the tactic once its mom was already performing her tactic and the calf's tactic was either the same as its mom's current or previously performed tactic within the observation period. Furthermore, we present observations of these calves in later years employing tactics they observed their moms perform. We conclude that the process of stimulus/local enhancement occurs in most of our observations and find evidence suggesting imitation in calves attempting to reproduce an opaque movement (i.e., movement with dissimilar sensory inputs when observed and executed) performed by its mom. These findings represent the first evidence of social learning and vertical transmission of foraging tactics in gray whales, suggesting that gray whales are capable of complex cognitive tasks and that foraging tactics may be part of PCFG gray whale culture.

## Graphical abstract

Opportunistic drone footage of 15 gray whale mom‐calf pairs collected over 9 years off the Oregon coast reveals evidence of social learning processes. This is the first evidence of imitation in gray whales and the strongest evidence for PCFG gray whale culture to date.

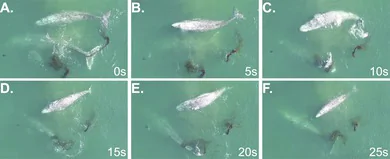

## 1 Introduction

Social learning, a key indicator of culture, can affect the behavioral diversity and ultimate survival of a population (Brakes et al. 2025). Given these significant effects, social learning and culture are increasingly recognized as valuable considerations in conservation (Brakes et al. 2019), especially given the prevalence of social learning across a diverse range of taxa (Whiten 2021). While culture is often identified based on the presence of social learning alone (Hersh et al. 2025; Brakes et al. 2025), characterization of the mimetic processes involved in social learning can provide valuable insight into a species' social structure and cognitive abilities (Whiten and Ham 1992).

The mimetic processes are classified by the different aspects of behavior that an observer copies from a demonstrator. Within the processes associated with social learning, the observer can learn the form (i.e., body movements; imitation) or goal of a behavior (goal emulation), what objects to use as part of a behavior (stimulus enhancement), and where (local enhancement) or when (observational conditioning) to employ a behavior (Whiten and Ham 1992; Heyes 2023). These processes can happen simultaneously and are thus difficult to identify and differentiate. Of the mimetic processes, imitation is of particular interest because of the potential association with cognitive abilities (Bates and Byrne 2010; Heyes 2023). Thus, the study of imitation in animals can further our understanding of its underlying cognitive mechanisms and their evolution across taxa (Heyes and Ray 2000). Furthermore, imitation strengthens social relationships (Harrison et al. 2024), is an inheritance mechanism for culture (Heyes 2023), and contributes to the cultural evolution of communicative and ritualistic behaviors (Heyes 2021). However, imitation is notoriously challenging to confirm (Whiten and Ham 1992; Miklósi 1999) and there are few documentations of its presence in the wild.

Most evidence of imitation in non‐human species comes from chimpanzees ( *Pan troglodytes* ), primarily in human care (Whiten and Ham 1992; Heyes 2021). Beyond primates, there is anecdotal evidence of motor imitation in fish (Mazeroll and Montgomery 1995), and experimental evidence of motor imitative capacity in banded mongoose ( *Mungos mungo* ) in the wild (Müller and Cant 2010) and odontocetes (i.e., toothed whales; bottlenose dolphins ( *Tursiops truncatus* ), orcas ( *Orcinus orca* ) and belugas ( *Delphinapterus leucas* )) in human care (Zamorano‐Abramson et al. 2023). Though imitation has not yet been documented in wild cetaceans (i.e., whales, dolphins, and porpoises), the prevalence of social traditions and learning (Whitehead 2018) in combination with experimental evidence (Zamorano‐Abramson et al. 2023) suggests its likely existence within cetacean species.

Cetacean behavior is especially difficult to observe in detail as they spend most of their lives underwater. However, the use of drones has revolutionized the field by providing a bird's‐eye view from which behaviors can be observed in greater detail for extended periods of time (Torres et al. 2018; Ramos et al. 2023; White et al. 2026). These advantages are especially true for cetaceans who spend time in shallow, coastal waters, such as the Pacific Coast Feeding Group (PCFG) gray whales ( *Eschrichtius robustus* ) who often feed in water less than 20 m deep (Bird et al. 2024a). The PCFG is a subgroup (~213 individuals; Harris et al. 2025) of the Eastern North Pacific population of gray whales (~17,750; Lang et al. 2026) that forages between Northern California, USA and Southern British Columbia, Canada (Calambokidis et al. 2019). PCFG gray whales employ a variety of foraging tactics that are not homogenously used; individual tactic use is associated with length (a proxy for age) and benthic habitat type (Bird et al. 2024a). These tactics target prey throughout the water column (e.g., benthic, mid‐water column, surface), range from stationary (e.g., headstanding) to forward swimming in various body orientations (e.g., side‐, upside‐down‐, and forward‐ swimming), and some tactics have been grouped by functional similarity (Bird et al. 2024a). While there is no evidence of tactics being socially learned, the high maternal recruitment of calves into the PCFG (Calambokidis and Pérez 2017) suggests social learning of space use. We hypothesize that during the short maternal period (~7 months from birth to weaning; Sumich 1986) calves socially learn foraging tactics from their mothers. Here we analyze opportunistic drone footage of PCFG gray whale mom‐calf pairs filmed foraging off the coast of Oregon, USA for evidence of foraging tactic mimicry and evaluate alignment with different mimetic processes.

## 2 Methods

### 2.1 Data Collection

Drone footage was collected during boat‐based surveys off the coast of Newport (44.60765, −124.08162), Oregon, United States between May and October of 2016–2025. Teams of 3–4 people conducted surveys *ad libitum* (Altmann, 1974) from a small (5.4 m) rigid hull inflatable boat in good weather (wind < 22 km/h, swell < 1.5 m, minimal fog or rain). The team approached whales upon sighting to collect photo‐identification images with DSLR cameras equipped with a 100–400 mm lens and conduct drone operations. The boat stayed at least 75 m away from the whale(s) during drone operations to avoid any effect on behavior. The pilot continuously recorded video during a flight (~15 min). The pilot located and followed the whale(s) while visible at the surface and underwater while maintaining the drone at an altitude between 20 and 40 m. Five different drones were used across the nine‐year study period: a DJI Phantom 3 Pro, 4 Advanced, and 4 Pro, an Inspire 2, and a Mavic 3 Pro.

### 2.2 Data Processing and Analysis

Individuals in drone footage were identified using aerial photo‐identification and matched to our long‐term catalog using concurrent lateral images. Footage was then clipped to times where a whale was visible. For this study we selected clips of mom‐calf pairs and of these calves in years following their birth year. A single experienced analyst (CNB) manually annotated all clips in the open‐source Behavioral Observation Research Interactive Software (BORIS; Friard and Gamba 2016) using an ethogram containing 10 behaviors (Table 1; Torres et al. 2018; Bird et al. 2024a). The analyst reviewed each clip at least twice per individual.

**TABLE 1:** Ethogram containing definitions of behaviors used in this study.

Group	Behavior	Definition
	At surface	Whale observed at the surface performing short breath hold dives in‐between blows.
	Near mom	Calf is observed near its mom (< 12 m) while the mom is engaged in a foraging tactic.
	Nursing position	Calf is observed going into a nursing position under its mother's ventral surface and positioning there for an extended period.
Surface tactics	Surface feeding	Whale feeding at the surface, frequently breaking the surface but without breathing. Characterized by frequent turning and frequent jaw snapping/flexing.
Forward swimming tactics	Side‐swim (forward)	Whale observed swimming on its side, moving forward. Characterized by frequent jaw snapping.
	Subsurface swim (forward)	Whale swims forward while feeding below the surface of the water oriented dorsal up. Characterized by frequent jaw snapping.
	Upside‐down swim (forward)	Whale observed swimming upside down, moving forward. Characterized by frequent jaw snapping.
Side‐swim (stationary)	Side‐swim (stationary)	Whale observed swimming on its side but not moving forward. Characterized by frequent jaw snapping.
Headstand	Headstand	Whale is positioned head down‐fluke up, or if in water depths less than whale body length (~12 m), whale may be more horizontal in water column; With both body positions, the whale is observed pushing head/mouth region into substrate through fluke beats and pectoral fin action.
	Body roll	Whale observed moving its body in a barrel roll.
	Bubble blast	Underwater release of air by whale that rises to surface and forms a circle/puka.

Mom‐calf pairs were first classified by whether the mom was observed performing a foraging tactic. As we had multiple clips of each mom‐calf pair, we then summed the clip durations per pair to obtain a total observation duration (hereafter “observation time”) and examined observation bias using a Mann–Whitney test to assess if pairs in which the mom foraged were observed for longer time periods than pairs with no foraging observed. Next, we focused on pairs in which both mom and calf performed a foraging tactic. For these pairs, we qualitatively compared the timing of the foraging dives and the tactics used by the mom and calf. Finally, we compared the tactics used by calves in later years to those they observed their moms performing as potential anecdotal evidence of vertical transmission. All processing and analyses were performed in R v.4.4.2 (R Core Team 2024). Data and code are available here: https://figshare.com/articles/journal_contribution/Data_and_code_for_Whale_See_Whale_Do_Drone_Observations_Reveal_Social_Learning_Processes_in_Mom‐Calf_Pairs_/31245571.

## 3 Results

Our opportunistic nine‐year dataset includes 15 mom‐calf pairs from 13 unique moms. Of the 15 mom‐calf pairs, we observed the mom performing a foraging tactic with her calf nearby in nine of the 15 pairs. In the six remaining pairs, we did not observe either the mom or calf foraging. We found a significant difference in observation duration between the two groups (*p* = 0.0076); the six pairs in which we did not observe the mom performing a foraging tactic were observed for less time (median = 3.44 min, SD = 3.43 min) than the pairs in which a foraging mom was observed (median = 13.93 min, SD = 5.96 min). These pairs that did not forage were observed either at the surface or nursing.

Of the nine pairs in which the mom was observed foraging, we observed six of the calves foraging as well. Interestingly, the three calves that we did not observe foraging spent most of their time while mom was feeding subsurface near their mom where we were unable to identify their behavior from the drone. Therefore, it is possible that these calves were observing or imitating their moms but we could not detect it. Of the six calves observed foraging, their foraging tactic always initiated after their mom began foraging (Figure 1). On average, calves initiated a tactic 13.2 s after their mom began foraging, but this timing was variable (range: 0.7–49 s, interquartile range: 4–19 s).

**FIGURE 1:**
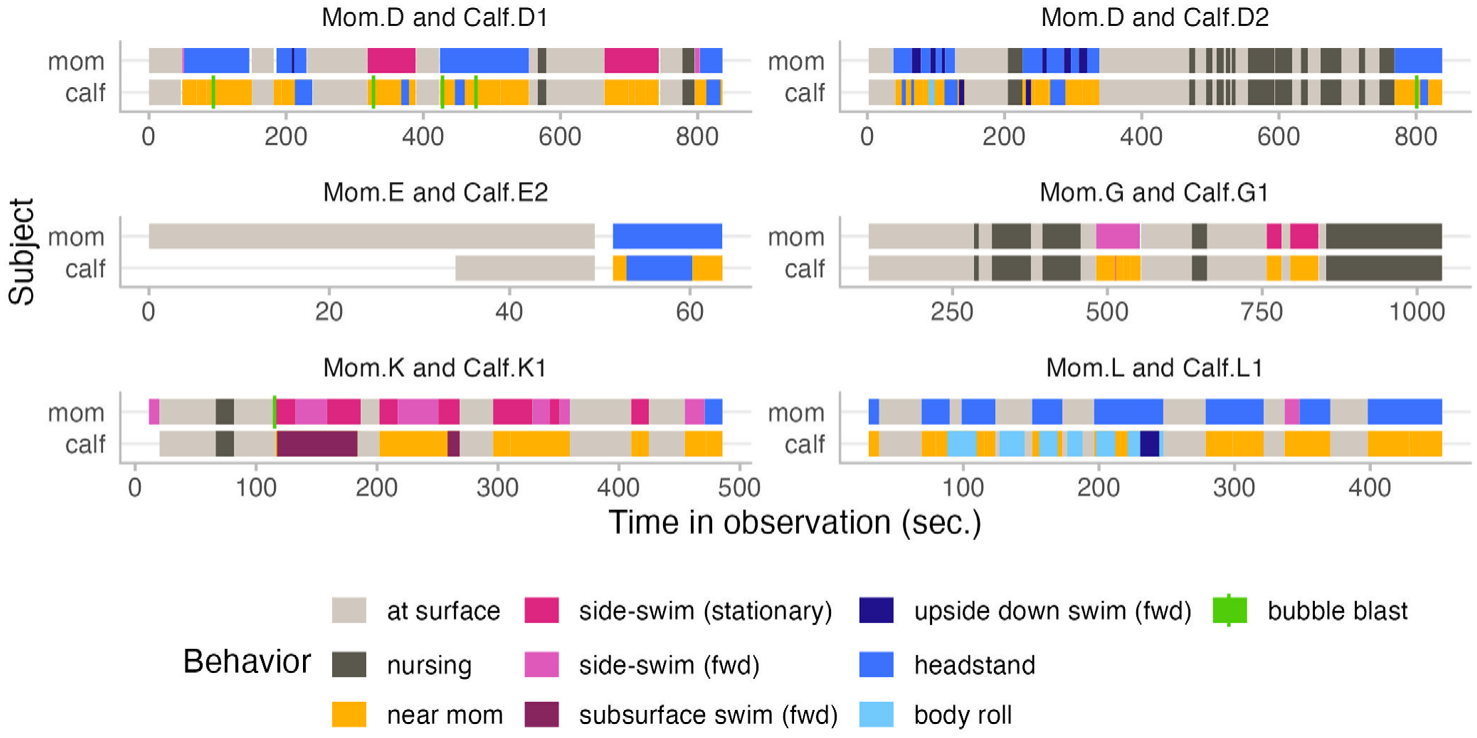
Behavior timelines for the six gray whale mom‐calf pairs where both whales foraged. Each facet represents a mom‐calf pair, with one timeline per individual, and colors represent behavior state.

Across the six calves observed performing a foraging tactic, we observed 21 foraging dives (total duration = 6.23 min). Calves performed the same tactic as their mom in nine of those dives (42.9%). In three of the 12 dives where tactic use differed, the calf used a tactic that its mom used earlier in the observation period, and in four other dives the calf used a tactic that is considered part of the same functional group as its mom's tactic (Bird et al. 2024a; Table 1). In the remaining five dives, the calf performed a body roll while its mom was headstanding (Figure 2), which we hypothesize is the result of the calf's unsuccessful effort to imitate its mom's headstand.

**FIGURE 2:**
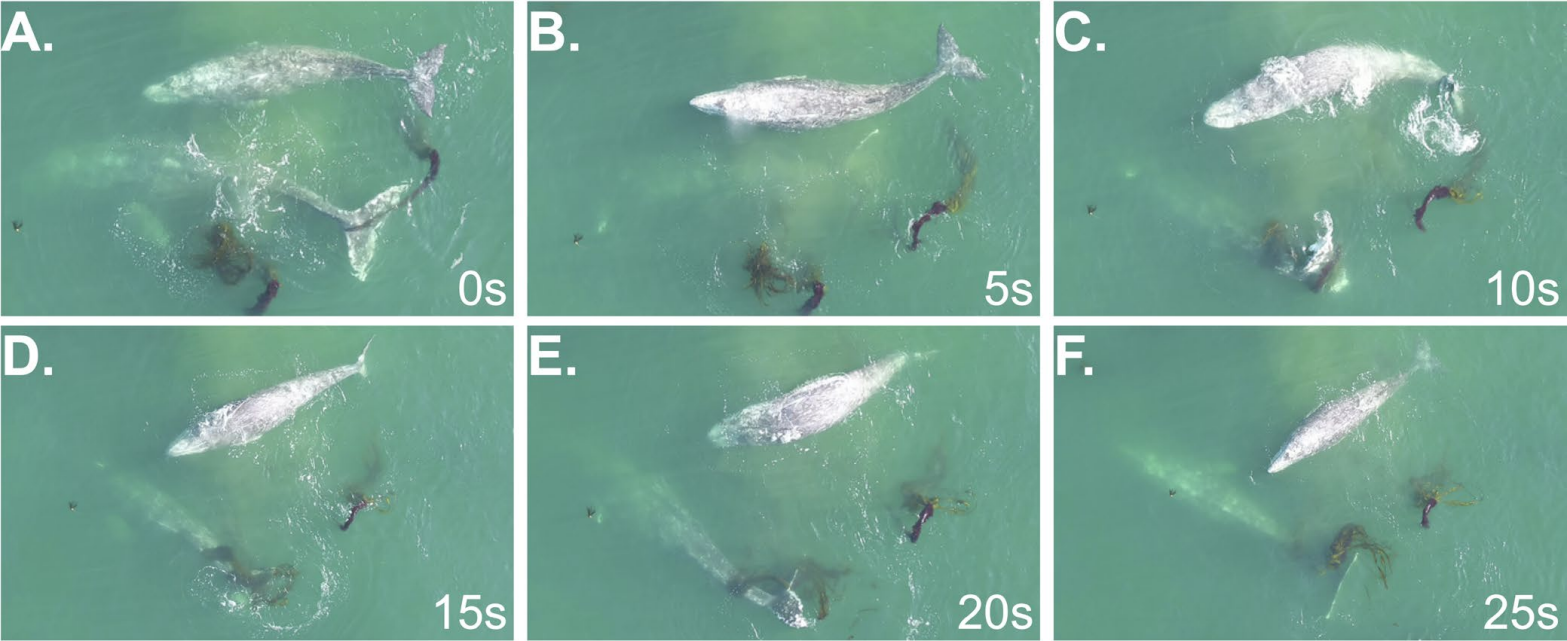
Sequential photos extracted from drone video in five‐second increments. Images A–F show Mom.L (whale in lower left region of frame) initiating a headstand while Calf.L1 performs a body roll. Note that in image D, both Mom.L and Calf.L1 are upside‐down.

We observed five of the 15 calves foraging independently in later years. Four of these calves were observed as 1–2‐year‐olds and used the surface feeding (*n* = 2) or forward‐swimming (*n* = 2) tactics. Both individuals observed forward‐swimming had observed their mom forward‐swimming as calves (Calves G1 and M1) and one had performed a forward‐swimming tactic while with their mom (Calf G1). The fifth calf (Calf D1 in Figure 1), who observed their mom headstanding and performed headstanding as a calf, was observed headstanding as a six‐year‐old.

## 4 Discussion

We posit that our observations of six calves performing foraging tactics while their mom was feeding may represent multiple social learning processes. The dives where the calf performed a tactic its mom had used earlier in the observation could represent instances of “delayed imitation”, which has been documented in bottlenose dolphins in human care (Xitco 1988; Zamorano‐Abramson et al. 2023) or “latent learning” that is associated with local/stimulus enhancement (Whiten and Ham 1992). It is difficult to distinguish local from stimulus enhancement in these observations because the calf could be learning to orient the feeding behavior to the prey or the habitat, or likely both. The dives where the calf performed a tactic from the same functional group as its mom's tactic are likely cases of enhancement, yet imitation may also have occurred as functional grouping means the tactic shared similarities in form with the mom's tactic. The dives where the calf used the same tactic as its mom may represent both enhancement and imitation, with a key unknown factor being if the calf already knew the tactic. If the calf already knew the tactic, then the calf was likely learning when and where to employ the tactic (i.e., enhancement). Imitation could have occurred if the calf was also learning how to physically perform the tactic. It's also possible that these are not cases of social learning but rather social influence (e.g., social facilitation/contagion; Whiten and Ham 1992), where the calf performs the tactic simply because it observes it's mom do so. However, our observations of calves performing tactics in later years after they observed their mom employ the tactics suggest that social learning is taking place.

Furthermore, we suggest that the body rolls performed by calves while their moms were foraging represent attempts to imitate a headstand. Body rolls do not align with goal emulation or enhancement as there was no apparent feeding during the behavior (e.g., no visible opening and closing of the mouth) and the calf was not diving close to where its mom was feeding (mom's head was close to the ocean floor while calf was at the surface). The physical complexity of a headstand suggests that body rolls are an effort to reproduce the opaque body movements of its mom (Heyes 2023). While headstanding is a common tactic performed by PCFG whales, this foraging tactic is used primarily by long mature whales and it is hypothesized to be a physically challenging behavior to perform that young individuals may struggle to accomplish (Bird et al. 2024a). When a whale headstands it dives into a steep pitch and roll (Colson et al. 2024), often times maintaining an upside down position while steeply pitched for long durations (Figure 2D; mean = 55 s, SD = 51 s; Bird et al. 2024b). The calves that performed body rolls while their moms were in a headstand, did not roll continuously, but rather paused mid‐roll in the upside‐down position (see video in FigShare repository). Given that body rolls were always initiated after mom's headstand, were only performed when mom was headstanding, and have rarely been observed in other contexts (*pers. obs*.), body rolls appear to be an imperfect (Whiten and Ham 1992) attempt by the calf to imitate the dive, pitch, and roll of a headstand.

The observations of calves foraging independently when older align with the documented ontogenetic shift in PCFG gray whales from the forward swimming tactics to headstanding (Bird et al. 2024a), as we only observed the 6 year old whale headstanding. But the potential presence of imitation as a calf shows that the calves are exposed to this tactic early in life and indicates that they begin to develop the skill from an early age. Furthermore, it is likely that social learning is not rare and occurs more frequently than observed, which is supported by our findings that (1) mom‐calf pairs observed foraging were observed for more time and (2) most calves who observed their moms foraging also foraged.

Notably, we also observed two calves performing bubble blasts (Figure 1). While these bubble blasts were not imitations, as the mom was not observed performing a bubble blast at that sighting, they may have already been exposed to bubble blasting as their mom had been observed bubble blasting in previous years (Bird et al. 2024b). These observations are interesting given the hypothesis that bubble blasts performed on the foraging grounds are an adaptation to regulate buoyancy during shallow foraging, particularly in fatter whales (Bird et al. 2024b). Interestingly, gray whale calves are relatively fatter than other demographic units (Lemos et al. 2020; Pirotta et al. 2023) and both calves observed bubble blasting were also observed headstanding, which is the foraging tactic during which bubble blasts are most likely to be performed (Bird et al. 2024b). Calves have also been observed performing bubble blasts in the wintering lagoons of Baja California Sur (*pers. obs*.). Thus, these observations may be an instance of local enhancement in which the calves learn the contexts in which bubble blasts are useful (Whiten and Ham 1992).

The potential presence of imitation and enhancement in these PCFG gray whales is the strongest evidence for these processes underlying social learning in baleen whales, and is the first evidence suggesting the presence of foraging culture in gray whales (Garland et al. 2025). This evidence is distinct from most cetacean studies where social learning of physical behaviors, and therefore culture, is detected using diffusion analysis (Hersh et al. 2025). Diffusion analyses have identified vertical transmission of the sponging foraging tactic in Indo‐pacific bottlenose dolphins ( *Tursiops aduncus* ; Wild et al. 2020) and cultural horizontal transmission of the lobtail foraging tactic in humpback whales ( *Megaptera novaeangliae* ; Allen et al. 2013). However, the specific mechanisms responsible for these instances of learning are unknown (Heyes 1994; Zamorano‐Abramson et al. 2023). Hence, our evidence that gray whales may be capable of motor imitation could indicate that gray whales possess advanced cognitive abilities and provides useful comparative information for questions regarding the evolution of these cognitive abilities across species. Furthermore, our findings may support future population management efforts as evidence of culture is increasingly used in cetacean population structure delineation and conservation (Brakes et al. 2019; Garland et al. 2025).

## Author Contributions

**Clara N. Bird:** conceptualization (equal), data curation (equal), formal analysis (lead), investigation (equal), methodology (lead), visualization (lead), writing – original draft (lead), writing – review and editing (lead). **Leigh G. Torres:** conceptualization (equal), funding acquisition (lead), investigation (lead), supervision (lead), writing – review and editing (equal).

## Funding

Data collection was supported by the NOAA National Marine Fisheries Service Office of Science and Technology Ocean Acoustics Program (2016 and 2017; 50‐27), the Oregon Sea Grant Program Development funds (2018; RECO‐40‐PD), the Oregon State University Marine Mammal Institute (2019), the Marine Mammal Commission (#MMC23‐146), and the Office of Naval Research Marine Mammals and Biology program (2020–2023: #N00014‐20‐1‐2760; 2025: #N000142412560).

## Conflicts of Interest

The authors declare no conflicts of interest.

## Data Availability

Data and code available from FigShare: https://figshare.com/articles/journal_contribution/Data_and_code_for_Whale_See_Whale_Do_Drone_Observations_Reveal_Social_Learning_Processes_in_Mom‐Calf_Pairs_/31245571.
